# Estimating the number of required nurses in different types of hospitals: An application of the workload indicators of staffing needs (WISN_S_) method

**DOI:** 10.1371/journal.pone.0295213

**Published:** 2023-12-14

**Authors:** Zhila Najafpour, Mohsen Zare Nasiri, Mohammad-Hussein Nozarian, Iman Keliddar, Kamran Shayanfard

**Affiliations:** 1 Department of Health care Management, School of Public Health, Ahvaz Jundishapur University of Medical Sciences, Ahvaz, Iran; 2 Department of Health Services Management, School of Public Health, Ahvaz Jundishapur University of Medical Sciences, Ahvaz, Iran; 3 Physics and Materials Science Research Unit, University of Luxembourg, Esch-sur-Alzette, Luxembourg; Bacha Khan College of Dentistry Mardan, PAKISTAN

## Abstract

**Background:**

Health system performance depends on the availability, accessibility, acceptability, and quality of health workforces. Policymakers seek whether the number of nurses is optimally matched based on patients’ needs. This study aimed to assess the workforce stock, workload activities, activity standards, and workload pressure to determine the number of required nurses in different types of hospitals in Iran.

**Methods:**

This study applied the workload indicators of staffing needs (WISN_s_) method and was conducted in 22 surgical and internal medicine wards at five hospitals in the southwest of Iran during six months. A time-motion study, and several group discussions, interviews were used to extract the required data. Descriptive statistics were used for data analysis.

**Results:**

All selected hospitals faced nursing shortages. The highest shortage (-47) and workload pressure (WISN ratio 0.45) were observed in the general-educational hospitals. In the specialized hospitals, the workload pressure was high (WISN ratio 0.49). The lowest shortage belonged to the private hospital. Based on our assessment, in all of the hospitals, nurses typically worked overtime due to high workload. The studied hospitals covered an average of 25% of their shortage with nursing overtime working. We noted that nurses were predominantly occupied with health service and supportive activities (≈90% of their time).

**Conclusions:**

Based on the WISN method, all of the hospitals faced nursing shortages from moderate to high. However, it would be essential to consider current labor market analysis based on accurate data to adopt appropriate policies in HRH planning.

## Introduction

Health system performance depends on the availability, accessibility, acceptability, and quality of human resources for health (HRH). According to the World Health Organization (WHO) reports, the lower density of HRH associates with poorer healthcare outcomes rates [1]. While, the substantial increase in the number of HRH has been associated with the higher the health sector expenditures [2]. Nurses, as the largest group of health providers, play a fundamental role in providing health services [3]. Therefore, nursing staff adequacy is consistently linked to the quality of care, patient outcomes, and patient satisfaction [4–6]. Then, HRH planners are always looking to determine appropriate nursing staff numbers and skill mix to meet hospital needs [7].

Based on the WHO’s National Health Workforce Accounts (NHWA) report in 2020, the global workforce stock was 29.1 million nurses. It is unequally distributed between high-income and low-income countries [8, 9]. So, one-third of the world’s population lacks access to health care because of HRH_S_ shortages. Clearly, a balance between the number and skill mix of HRH and health needs is a significant challenge to achieve Universal Health Coverage (UHC) and the Sustainable Development Goals (SDG_S_), especially in less developed countries [10].

Various tools have been established to calculate the number of required HRH. The WHO’s Workload Indicators of Staffing Need (WISN) method is one of the practical tools to improve processes for planning HRH. Based on the WISN, HRH needs are determined based on the experienced workload by health facilities. In other words, this technique considers the number and skill mix of HRH currently available, the time that they are available, and the amount of works each one can accomplish during this available time. The WISN has eight steps, including determining priority cadres; estimating available working time yearly; defining workload components; setting activity standards; establishing standard workloads; calculating allowance factors; determining staff requirements based on the WISN; and analyzing and interpreting WISN results [11].

The WISN application helps HRH planners to identify the shortage or excess of human resources based on the workload pressure. Based on the evidence Iran, like many other countries, faces a shortage in the number of nurses [12]. Based on our knowledge, there is not any rigorous technique to determine the size and skill mix of HRH in Iranian health care system. Regarding the mentioned situation, this study aimed to assess the workforce stock, workload activities, activity standards, and workload pressure to determine the number of required nurses in hospitals.

## Material and methods

**Fig 1:**
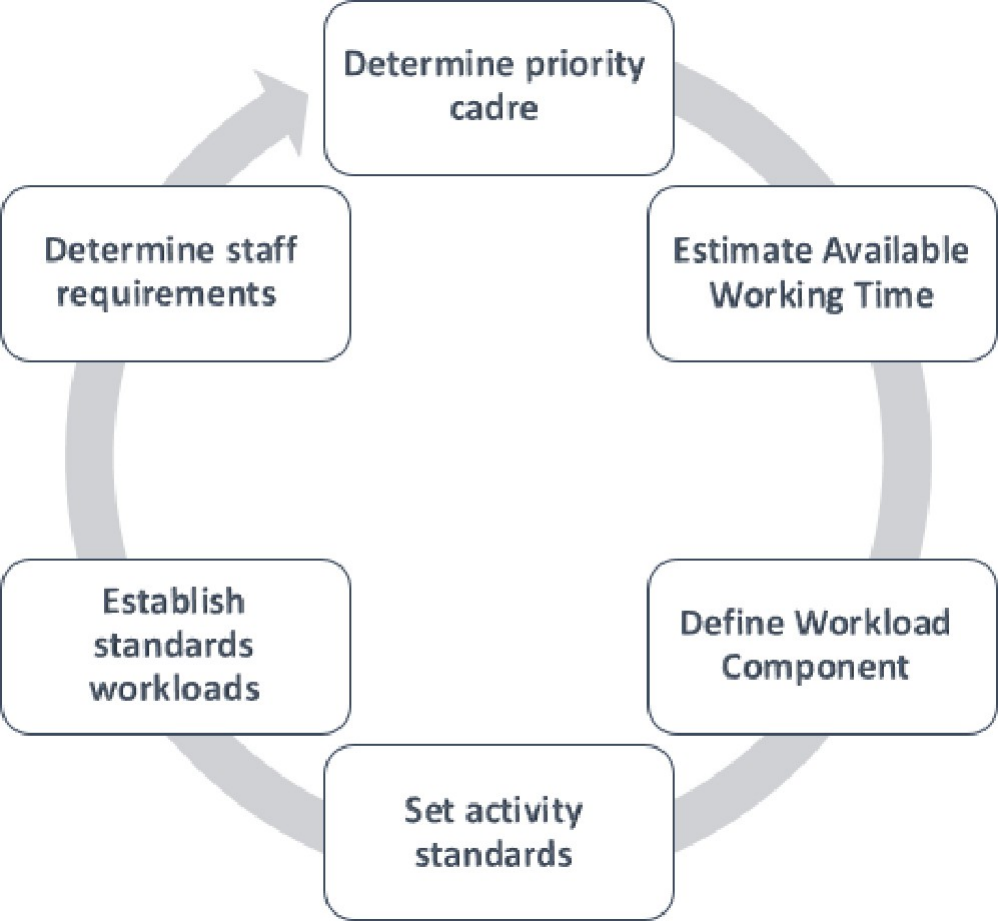
The WISN steps.

This study has been carried out with mixed methods design, combining expert panels, interview, and an observational study to define and quantify the nurses’ workload components between February 1^st^ and August 1^st^ 2022. We used the WISN technique in surgical and internal medicine wards of five public and private hospitals in the southwest Iran. Fig 1 shows the WISN steps.

1. **Determine priority cadre(s):** Firstly, we listed the main categories of health workers at hospitals in Iran. After that, participants were selected based on their involvement in HRH management in the health system. Three hospital managers, two academicians, three HRH managers, and two quality improvement experts participated in this part. The participants determined the highest priorities based on the questions "Which staff category is in the shortest supply based on need criteria?" and "Which of these staffing problems have the most impact on the quality of care?". The priority point scale was between 0 (without priority) to 10 (the highest priority) (see Table 1).
2. **Estimate Available Working Time (AWT):** In this step, we calculated the total number of working hours available for each HRH in the previous year of the study (2021) using the formula AWT = [A (B + C + D + E)] *F. In this formula, A is the total number of working days per year, B is the number of public holidays, C is the number of annual leaves, D is the number of sick leaves, E is the number of other leaves, and F is the number of working hours per day.
3. **Define Workload Component**: In this part, we used an expert panel, interview, and an observational study to define and quantify the workload components. Nursing activities were identified and classified based on the heterogeneity in the studied wards using the WISN tool [2]. Firstly, an initial list of nursing care activities was formulated based on reviewing the literature and observing nursing care activities in several wards in the selected hospitals. The first draft of nursing activities was finalized based on the Nominal Group Technique (NGT). The initial list was referred to an expert panel (15 nurses) to confirm the activities’ categorization.
4. **Set activity standards:** This step describes standard workload components conducted by a well-trained skilled worker per year. Data collection processes were continued to reach data saturation in the time of activities. After that, we calculated the average of the recorded times and confirmed the standard times acquired by several expert panels (n = 25 nurses) in the studied wards. In terms of workload components, because of the lack of electronic patient records in the studied hospitals and the low quality of recorded data, we decided survey to count the nursing care time per shift.
  **4.1. Health service activities**: To calculate the time of health service activities, a prospective and observational time and motion study was performed. This part was conducted in 24 internal medical and surgical wards in five public and private hospitals over six months in 2022. A stopwatch was used, minutes were used as the unit for work time measurement, then converted to hours. The workload included all of the activities that were done by nurses. Nursing activities were the unit of sampling in this study. After informed consent had been obtained, two observers shadowed each nurse during working shifts. During the study, observers attended in wards for the 7–12 hours of working shifts (morning and evening shifts). Times of observation were determined according to a predefined schedule. Each ward was observed for a minimum of two weeks. Inter-rater reliability was tested by collecting data simultaneously. An inter-rater agreement test was done to check the reliability of the collected data. For this reason, a third observer temporarily joined the main observers. Meanwhile, we determined inter-rater agreement as the percentage of the same agreement with less than five percent differences. A trained researcher observed all activities done by nursing staff in different working shifts for two days to verify the quality of the collected data. In low-frequency cases, a nurse interviewee was also conducted. Every activity performed by each nurse was directly observed by our assessors on ten random occasions resulting in a minimum of 30 observations for each activity. Descriptive statistics (means, frequencies, percentages) were used for data analysis.
  **4.2. Allowance standards**: An allowance standard is an activity standard for support and additional activities. The time of support activities was calculated based on observations in the previous phase. Since it was impossible to directly observe additional activities, the time of these activities was calculated based on approximations obtained from nurses during interviews (n = 15). Category allowance standard (CAS) is the activity standard for support activities performed by all staff members of a cadre. CAS is calculated as the percentage of working time spent on each support activity. Category allowance factor (CAF) is the required number of nurses for health service and support activities that calculated using the following formula, CAF = 1/ [1 - (Total CAS/100)]. Individual allowance standards (IAS) were obtained by calculating how much time additional activities of certain cadres require.
5. **Establish standards workloads:** The service standards represent the possible volume of work that a nurse conducts per year. It was calculated by dividing the AWT in a year by the unit time for each activity.
6. **Determine staff requirements:** The number of required staff is obtained for each activity by dividing the annual workload of each activity by the standard workload of the same activity. The total number of required nurses is calculated by multiplying the total staff required with the CAF and then adding the individual allowance factor to the result. Finally, we analyzed the results in terms of difference. According to the WISN toolkit, we categorized the workload pressure based on the WISN ratio as extremely High (0.10 and 0.29), very high (between 0.30 and 0.49), high (between 0.50 and 0.69), moderately high (between 0.70 and 0.89), normal (between 0.90 and 1.19) and low (greater than or equal to 1.20).

**Table 1:** Priority score cadre.

Staff category	Score (Mean)
Physician	5.5
Nurse	7.1
Midwives	5.2
Radiography Technologist	3.8
Laboratory Technologist	3.8
Pharmacist	2.9
**Highest Priority score cadre: Nurse**	

### Data analysis

All extracted data was checked and entered into the Excel software and analyzed based on the WISN protocol.

## Results

General information on five selected hospitals is shown in Table 2. Based on our assessment, nurses were the highest priority score cadre. The number of nurses in each ward varied among hospitals (10–31 nurses in each ward).

**Table 2:** General information of five hospitals.

	General hospitals		specialized hospitals		Private hospital
	Hospital A	Hospital B	Hospital C	Hospital D	Hospital E
Number of beds	720	684	269	400	233
Number of wards	18	17	14	13	7
Number of observed wards	5	6	7	2	4
Bed occupancy rate	80%	78.5%	64%	63%	73%
Average of length of stay (LOS)	6.3	5.7	3.81	3.7	3.5

In general, the available working time (AWT) of hospitals was 1437 hours per year (see Table 3).

**Table 3:** Calculating nurse’s available working time (AWT).

Variables	Calculation
Number of days of the year	365
Number of public holidays	27
Number of Fridays	52
Annual Vacation and Sick leaves	30
Training hours Per Year	80
Working hour per week (h)	44
Working hours (annually)	1437

Table 4 demonstrates the standards for nursing activities. On average, nurses spend 47.6 min for each patient per shift on health service activities. Allocated time in the specialized hospitals was more than in other hospitals (63 min). The findings showed that the allocated time for health service activities varied. For example, allocated time in specialized hospitals for infusion intravenous fluids and serum therapy was more than others (4.5 vs 3 min). Chemotherapy as a specialized activity took 17 min on average for each patient. The required time for wound dressings in private hospitals was more than in others (4 vs. 2.5 min). While some activities like medication administration and checking of vital signs had similar times.

**Table 4:** Health service activities.

	Workload component	Annual workload			Standard workload			Required number of staff members		
		General hospitals	specialist Hospitals	private Hospital	General hospitals	specialist Hospitals	private Hospital	General hospitals	specialist Hospitals	private Hospital
**Health service activities**	infusion of intravenous fluids, serum therapy, and blood transfusion*	168480	131976	131976	28740	19160	19160	5.86	6.89	1.66
	Collecting, labeling and dispatch of laboratory samples	265824	174720	174720	21555	17244	17244	12.33	10.13	2.56
	Chemotherapy management	438984	276120	0	12317	11496	0	35.64	24.02	0.00
	Drug preparation and administration	128700	78624	276120	34488	24634	11496	3.73	3.19	7.23
	Wound Dressings	106740	72384	78624	28740	21555	24634	3.71	3.36	1.97
	Patient self-care management, e.g., education, counseling patients, emotional supports	66456	34944	72384	86220	86220	21555	0.77	0.41	1.38
	Observing vital signs and input-output	126360	87360	34944	17244	12317	86220	7.33	7.09	0.16
	Transferring patients to other hospitals, wards, scan or operating room	35568	27144	87360	86220	57480	12317	0.41	0.47	4.98
	Performing hygiene procedures	92664	67392	27144	28740	21555	57480	3.22	3.13	0.34
	Accompanying the doctor’s round	71136	36192	67392	86220	86220	21555	0.83	0.42	1.18
	Requesting lab tests, imaging, blood reservation, so on	73008	53040	36192	43110	34488	86220	1.69	1.54	0.17
	Follow-up the patient’s paraclinical procedures	92664	75816	53040	28740	19160	34488	3.22	3.96	0.34
**Total required staff for health service activities**								**78.78**	**64.60**	**18.43**

Table 5 shows the time spent on supportive and additional activities for three types of hospitals. The results showed that the total spending time of supportive activities varied from 35 min in the specialized hospitals to 42 min in the private hospital. Supportive activities included patient handover (4.8 min), documentation and reporting (25.3 min), and patient communication (mean 4 min) per patient. The maximum time for additional activities was 272 hours of the total working time of a nurse per year that belongs to the specialized hospitals. It includes ward meeting sessions, educational courses, student supervision, training students, and other staff.

**Table 5:** Category allowance standards and individual allowance standards.

**Support activities**	**Workload component**	**CAS (Actual working time) (percent)**		
		**General hospitals**	**specialist Hospitals**	**private Hospital**
	Patient handover	4 (0.95%)	4.5 (1/07%)	6 (1.43%)
	Documentation and reporting	26 (6.19%)	23 (5.48%)	27 (6.43%)
	Maintaining relationship with patients, their relatives, and colleges	3 (0.71%)	4 (0.95%)	5 (1.19%)
	Checking medical Emergency Trolley and equipment incidence reporting and patient safety related affaires	4 (0.95%)	4 (0.95%)	4 (0.95%)
**Total CAS percentage**		**8.81%**	**8.45%**	**10.00%**
**Category allowance factor: {1 / [1 - (total CAS percentage / 100)]}**		**1.096**	**1.092**	**1.099**
**Additional activities**	**Workload component**	**Annual IAS (for all staff performing activity)**		
		**General hospitals**	**specialist Hospitals**	**private Hospital**
	Meetings	24h per year	24 per year	0
	Education courses	80h per year	80per year	40 per year
	Students’ supervision	96h per year	108 per year	0
	Training students and other staff	48h per year	60 per year	0
**Total IAS in a year (h)**		**248**	**272**	**40**
**Individual allowance factor (Annual total IAS / AWT)**		**0.17**	**0.19**	**0.03**

Additionally, CAF includes the required staff for both health service and support activities (range: 1.092–1.099), and IAF includes required staff to cover additional activities calculated (range: 0.03–0.19) that were not large. They did not lead to a significant difference in the final total number of required nurses. Some activities, including clinical rounds with the supervisor and performing accreditation activities, and registering information and statistics, were conducted by the head nurses. Additionally, in our assessment, we found several patient care services that were provided by health care assistants and patient relatives (i.e., bringing a collection of samples to the laboratory, patient hygiene, changing the position of patients, toileting aids, moving a patient from bed to wheelchair and reverse. However, we could not extract time for the mentioned activities. We did not consider the mentioned activities.

Table 6 presents the required number of nurses for each type of hospital using the WISN tool. All selected hospitals faced nursing shortages. The highest shortage (-47) and workload pressure (WISN ratio 0.45) are observed in the general hospitals. The results showed that the required nurses in general educational hospitals based on the workload pressure is 88. In the specialized hospital, the workload pressure is high (WISN ratio 0.49). The lowest shortage belongs to nurses in the private hospital with an average deficit of eight nurses. Based on our assessment in all of the hospitals, nursing staff worked overtime. However, in the hospital working overtime was not obligatory. However, nurses are required to work more than their regularly scheduled 40-hour week since the nursing shortages. Nurses worked overtime in general hospitals, specialized hospitals, and the private hospital an average of 74 h, 62 h, and 41 h each month, respectively. After considering overtime hours, the required number of nurses has changed. Then, the studied hospitals covered an average 25 percent of their shortages with overtime working. Meanwhile, we found no nurses who worked longer than 12.5 consecutive hours a day.

**Table 6:** Required number of nursing staff.

Health center		Observed wards	Required number of nurses based on current number of nurses						Required number of nurses based on FTE				
			Current number of nurses	Required number	Shortage or excess	Workforce problem	WISN ratio	Workload pressure	Number of FTE	Shortage or excess	Workforce problem	WISN ratio	Workload pressure
Public hospitals	General	11	39	88	-47	Shortage	0.45	Very High	59	-27	Shortage	0.69	High
	Specialized	9	35	71	-36	Shortage	0.49	Very High	56	-15	Shortage	0.79	Moderately High
Private hospital		4	12	20	-8	Shortage	0.60	Very High	16	-4	Shortage	0.80	Moderately High

## Discussion

We followed the steps of WHO’s WISN methodology to assess the current workload and staffing need for delivering health care in the public and private sectors in Iran. based on our results, hospitals faced an overall shortage in nursing staff from moderately high to high. The studied hospitals covered an average of 25% of their shortages with working overtime.

The need for an evidence-based planning that could estimate actual HRH needs in health facilities is principal. Based on the literature, approaches to HRH estimation can be categorized into several metrics, including headcount, HRH numbers per population or bed, full-time equivalent (FTE) [13], and WISN [11]. The WISN is recommended to determine the optimal number and skill mix of HRH considering limited resources or constraints in different health facilities. The WISN method calculates the required staffing levels in different contextual. It emphasizes on service utilization rates, the daily activities conducted by health workers, and the time expended in service delivery.

Nurses, as the largest group of health providers, play a fundamental role in providing health services [4]. According to our results, staffing levels are not adequate to provide the expected services and to handle the accompanying workload. All studied hospitals faced nursing shortages. According to a recent study, the health system in Iran faces nursing shortages because of low social status, work-related injuries, early retirement, immigration, willingness to abandon the current job, employment in other professions, housekeeping, low employment rates, and the increase of hospital beds. Additionally, lack of balance in the proportion of workload with salary is one of the main reasons of nursing shortages [12]. We found the number of nurses is not optimally matched to the hospital wards based on patients’ needs. Our findings confirmed similar findings of nursing shortages [14, 15]. However the nursing shortages differed based on different hospital types [16, 17].

In response to high workload pressure and nursing shortages, several solutions are conducted by nursing managers, like using nursing assistants and midwives in nursing job positions [18]. Meanwhile, in the studied hospitals some supports where workload of a staff category was ‘extremely high’ were conducted. For example, nurses from lower workload wards were transferred to higher workload ones. Additionally, task shifting is a policy option when the health system faces to health workforce shortages and skill mix imbalances. Based on our assessment, 25 percent of the hospital shortages are covered with added nursing mandatory overtime.

Our findings demonstrated that the AWT of hospitals was 1437 hours per year. We did not have any difference in the hours worked among hospitals annually. Although Nguyen et al., reported a substantial difference in the hours worked by nurses between hospitals and between the departments of the same hospital in Vietnam [19].

We considered activity standards using direct observations and time-motion method. It is confirmed that nurses would spend most of their working shift’s hours beside the patients to provide direct care. Based on the results, nurses spend about 90 percent of their time on patient-related care activities, including health services and supportive activities per shift. We find an unequal workload across different hospitals and settings. So that the highest workload belonged to the general-educational hospitals. Recent evidence has contradicted results. A study showed that the maximum of nurses’ time spent on administrative and paperwork tasks [20]. Another study reported that support activities account for 31% of nurses’ workload. Studies have reported substantially lower times when nurses document electronically compared to paper [21]. Our studied hospitals used only paper charts, and nurses allocated seven percent of their time for recording per patient. In comparing paper and electronic nursing documentation, previous studies indicated that technology might reduce the nurses’ workload [22, 23]. Additionally, in response to nursing shortages, some of the nursing tasks were conducted by the nursing assistant cadre or patients’ families.

Our study faces several limitations. One of the limitations was that the study conducted in five hospitals; it might be limited the result’s generalization to other hospitals and settings. Although in this study, we selected different hospital settings (public and private), and wards (internal medicine and surgical) to apply and to modify easily for different settings and specialties. Different specialties have different working processes; in the study surgical and internal medicine wards are included to cover the differences. We considered nurses who provide direct care to patients in nursing positions, then head nurses did not include in our sample. Meanwhile, we did not consider nurses activities in ICUs and night shifts due to the large differences.

## Conclusions

This study provides significant evidence to help policymakers to determine HRH norms to meet patients’ needs in different types of hospitals. All selected hospitals faced with some degree of nursing shortages. The general hospitals had the highest shortage rate and workload pressure. The lowest shortage belonged to the private hospital. The WISN technique, as a practical method, helps HRH planners to identify the shortage or excess of human resources based on the workload pressure. It would be essential to consider current labor market analysis based on accurate data to adopt appropriate policies in HRH planning.

## List of abbreviations

WISN: Workload indicators of staffing need
HRH: Human resources for health
WHO: World Health Organization
NHWA: National Health Workforce Accounts
UHC: Universal Health Coverage
SDG: Sustainable Development Goal
AWT: Available Working Time
NGT: Nominal Group Technique
CAS: Category allowance standard
CAF: Category allowance factor
IAS: Individual allowance standards
FTE: Full-time equivalent
